# Ebp1 expression in benign and malignant prostate

**DOI:** 10.1186/1475-2867-8-18

**Published:** 2008-11-24

**Authors:** Philippe O Gannon, Ismaël Hervé Koumakpayi, Cécile Le Page, Pierre I Karakiewicz, Anne-Marie Mes-Masson, Fred Saad

**Affiliations:** 1 Centre de recherche du Centre Hospitalier de l'Université de Montréal (CR-CHUM) and Institut du cancer de Montréal. 1560 rue Sherbrooke East, Montreal, Quebec, Canada; 2 Cancer Prognostics and Health Outcomes Unit & Department of Surgery, Hôpital St-Luc (CHUM), 1058 rue St-Denis, Montreal, Quebec, Canada; 3 Department of Medicine, Université de Montréal, Montreal, Quebec, Canada; 4 Department of Surgery, CHUM, Université de Montréal, Montreal, Quebec, Canada

## Abstract

**Background:**

ErbB3-binding protein 1 (Ebp1) is a member of the *PA2G4 *family of proliferation-regulated proteins that is expressed in multiple malignant and non-malignant cells. ErbB3 and other members of the EGFR family have been implicated in cancer progression, it however remains unknown whether Ebp1 participate in prostate cancer progression *in vivo*. Therefore, the present study examines Ebp1 expression in cancerous and non-cancerous prostates tissues. Ebp1 expression was also correlated to known Ebp1 regulated proteins (Androgen receptor (AR), Cyclin D1 & ErbB3) and the proliferation marker Ki67. Furthermore we evaluated whether Ebp1 expression correlated with biochemical recurrence (BCR) following radical prostatectomy.

**Methods:**

The expression of Ebp1, AR, Cyclin D1, ErbB3 and Ki67 were evaluated by immunohistochemistry using three separate tissue micro-arrays containing normal prostate tissues, non-cancerous tissue adjacent to the primary tumor, hormone-sensitive and hormone-refractory cancerous tissues. Multivariate COX regression analysis was performed with four clinical parameters in order to correlate Ebp1 expression with PCa progression.

**Results:**

The expression of Ebp1 significantly increased with the progression from normal to hormone sensitive and to hormone refractory PCa. Furthermore, we observed strong correlation between Ebp1 expression and the nuclear expression of AR, Cyclin D1 and ErbB3 in both normal adjacent and cancer tissues. The expression of AR, Cyclin D1 and ErbB3 in normal adjacent tissues correlated with PSA relapse, whereas Ebp1 on its own did not significantly predict PSA relapse. Finally, in a multivariate analysis with a base clinical model (Gleason, Pre-op PSA, surgical margins and P-stage) we identified the multi-marker combination of Ebp1+/Cyclin D1- as an independent predictor of PSA relapse with a hazard ratio of 4.79.

**Conclusion:**

Although not related to disease recurrence, this is the first *in vivo *study to report that Ebp1 expression correlates with PCa progression.

## Background

Prostate cancer is a major public health concern in North American and European countries. Through improvements in diagnostic methods and increased awareness, greater than 90% of patients are now being diagnosed with localized or regionally advanced prostate cancer [1]. Nonetheless, between 15% and 35% of patients will develop prostate specific antigen (PSA) relapse within 10 years following surgery [2,3]. As the course of the disease is quite variable, progress needs to be made in the establishment of clinical tests that could help identify patients at risk of recurrence and progression. The molecular characterization of tumor cells through immunohistochemical analysis offers the opportunity to better stratify patients early in the disease process.

We and other groups have recently demonstrated an association between the nuclear localization of ErbB3, an epidermal growth factor receptor, and prostate cancer progression [4-6]. A protein directly interacting with ErbB3 and known to transduce growth regulatory signals is ErbB3-binding protein 1 (Ebp1). Cytoplasmic Ebp1 associates with the cytoplasmic domain of ErbB3 and following heregulin activation of ErbB3, Ebp1 dissociates from the receptor and translocates to the nucleus [7].

Ebp1 is a member of the *PA2G4 *family of proliferation-regulated proteins that is ubiquitously expressed in the cytoplasm and nucleus of multiple malignant and non-malignant cells [7,8]. Ebp1 activity may be involved in tumorigenesis through its roles in cellular proliferation and regulation of gene expression. In breast and prostate cancer, as well as in human fibroblasts, the ectopic expression of Ebp1 can limit cellular proliferation and promote differentiation [9,10]. Ebp1 can also repress the transcription of androgen receptor (AR) and Cyclin D1, through physically binding to the AR or by interacting with the histone deacetylase HDAC2 and the tumor suppressor Retinoblastoma (Rb) [10-13].

There are currently few studies describing the expression of Ebp1 in tumor tissues and its clinico-pathological relevance. Ebp1 is expressed in prostate and breast cancer primary culture [8]. A recent publication also showed an overexpression of Ebp1 in colorectal cancers compared to normal areas adjacent to the cancer [14]. To our knowledge the present study is the first to characterize the expression of Ebp1 in prostate cancer patients. Considering the important role of the AR in prostate cancer progression and possible regulation of AR expression by Ebp1, we deemed it necessary to evaluate the co-expression of Ebp1 with nuclear AR. Furthermore, since Ebp1 is involved in cellular proliferation, we also evaluated the co-expression of Ebp1 with two proliferation markers, Cyclin D1 and Ki67. Finally, the interaction between Ebp1 and ErbB3 was also studied. This report offers direct evidence that the high expression of Ebp1 correlates with the presence of prostate cancer as well as a double marker combination that includes Ebp1 that can help predict PSA relapse following radical prostatectomy.

## Methods

### Patient cohort

Three different tissue micro-arrays (TMAs) were used in this study. The first TMA contained 39 normal prostate specimens obtained from patients autopsied and will be referred to as the normal-TMA (N-TMA). A second TMA contained tumor tissue obtained by trans-urethral resections of the prostate (TURP) from 36 hormone refractory patients collected subsequent to hormone therapy failure. This second TMA will be referred to as the hormone-refractory TMA (HR-TMA). Finally, 62 patients who had undergone radical prostatectomy were selected for the creation of a third TMA based on the following inclusion criteria: no preoperative hormone therapy, follow-up of a minimum of 5 years or until death and received no radiotherapy or hormone therapy prior to biochemical failure [15]. This third TMA will be referred to as the radical prostatectomy TMA (RP-TMA). This sub-cohort of 62 hormone sensitive patients was used to evaluate the prognostic value of the molecular markers used in this study. The failure interval was defined as the time between radical prostatectomy and the first PSA reading above 0.2 to 0.4 ng/ml [14]. There was no age difference between the group of patients that develop PSA relapse and the group that did not. Clinical and pathological characteristics of the patients selected for the RP-TMA are presented in Table 1. The final pathological staging and grading was based on pathology reports from the CHUM Hôpital Notre-Dame. Ethics approval for this study was obtained from the local IRB committee and all patients signed consent forms.

**Table 1:** Clinical and pathological characteristics of patients in the RP-TMA.

Number of patients selected	62
Age median (min-max)	62 (49 – 70)

Pathological Stage	
*Stage 2*	34 (54.8%)
*Stage 3*	29(46.8%)

Invasion	
*Extracapsular*	19(30.6%)
*Lymph node*	9(14.5%)
*Perineural*	9(14.5%)

Positive surgical margins	32(51.6%)

Prostatitis	1 (1.6%)

Gleason score	
*Gleason 4*	8 (12.9%)
*Gleason 5*	14 (22.6%)
*Gleason 6*	13 (21.0%)
*Gleason 7*	17(27.4%)
*Gleason 8–9*	10(16.1%)

Pre-operative PSA	
<*10 ng*	35 (56.5%)
> *10 ng*	25 (40.3%)
*not available*	2 (3,2%)

PSA relapse	35 (56.5%)

Hormone refractory disease	5(8.1%)

Overall survival	54(87.1%)

Clinico-pathological parameters of the 62 patients selected for the construction of the RP-TMA.

### Tissue micro-array

We have previously described in detail TMAs obtained from normal prostate tissues and hormone refractory TURP specimens [16]. The third RP-TMA was built in duplicate and contained a total of 2 normal adjacent and 4 tumor 1.0 mm cores per patients for all 62 patients [15,16]. Two pathologists evaluated H&E staining of each TMA and confirmed the proper histological status each core.

### Characterization of normal adjacent tissue

**Figure 1:**
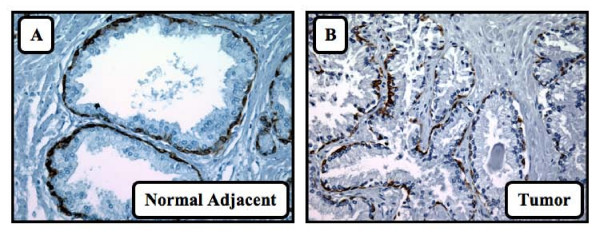
**Expression of cytokeratins in normal adjacent and tumor tissues**. Staining of the basal membrane with the cytokeratins Eβ12 antibody. A) Staining of a normal adjacent tissue with proper basal membrane integrity and continuity. B) Staining of a tumor tissue without proper basal membrane integrity and continuity.

Each core classified as normal adjacent was reviewed independently by two pathologists in a blinded manner. None of the normal adjacent cores contained histopathological evidence of neoplasia. To ensure that the normal adjacent cores were not cancerous and did not contain observable pre-neoplastic lesions, we evaluated the integrity and continuity of the basal membrane. By immunohistochemistry using an antibody that recognizes cytokeratins 1, 5, 10 and 14, we were able to distinguish between adenocarcinoma and non-adenocarcinoma of the prostate (Figure 1) [17]. From the 62 patients represented on the RP-TMA, 9 patients were removed because their corresponding normal adjacent cores did not satisfy our inclusion criteria, two of which developed biochemical recurrence. Statistical analyses were thus based on 53 patients for marker expression in normal adjacent tissues and 62 patients for marker expression in tumor tissues.

### Immunohistochemistry

Immunohistochemical staining of the prostate cancer TMAs was done as previously described [4,15]. Briefly, TMAs were deparaffinized in toluene and rehydrated in a gradient of ethanol. Endogenous peroxidase activity was quenched during a 30 min incubation with 0.3% H_2_O_2_/methanol. A 15 min incubation in boiling citrate solution (10 mM, pH 6.0) followed by a 15 min incubation at room temperature was used for antigen retrieval. Subsequently, a protein blocking serum-free reagent (Dako Cytomation Inc., Mississauga, Ontario, Canada) was applied for 15 min. The 60 min primary antibody incubation was completed in a humidified chamber with the following antibodies: Androgen receptor (Ab1, Labvision Neomarkers, CA, USA), Cyclin D1 (Ab4, Labvision Neomarkers), Cytokeratin EβE12 recognizing cytokeratins 1, 5, 10 and 14 (Chemicon, CA, USA), Ebp1 (07-397, Upstate, Lake Placid, NY, USA), ErbB3 (Sc-285, Santa Cruz biotechnology, CA, USA) and Ki67 (SP-6, LabVision NeoMarkers). The optimal dilution for each antibody was determined by serial dilutions. After stringent washes in PBS, the TMAs were incubated for 20 min with a secondary biotinylated antibody followed by 20 min incubation with a streptavidin-peroxidase complex (both from Dako Cytomation). Positive signal was developed with diaminobenzidine (DAB) (Dako Cytomation) and the nuclei were counterstained with haematoxylin.

### Interpretation of immunohistochemistry results

**Figure 2:**
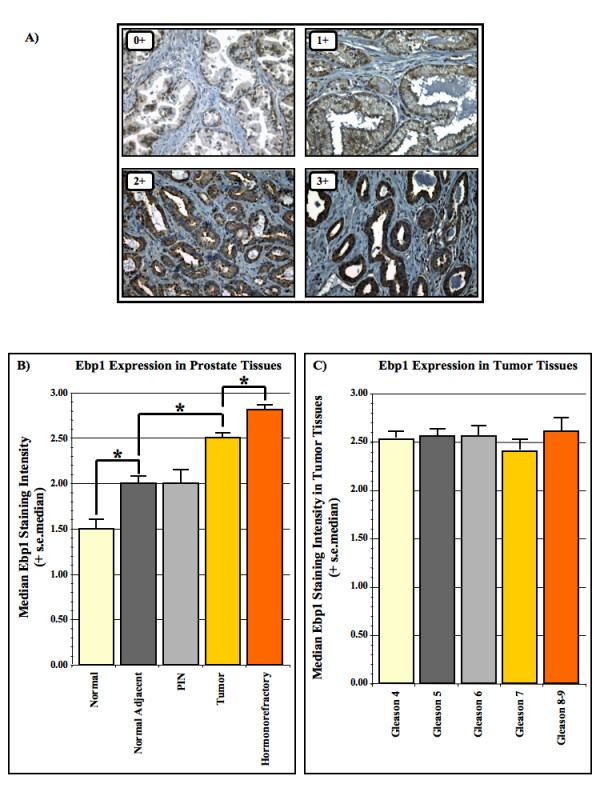
**Expression of Ebp1 in prostate tissues**. A) Prostate epithelium stained for Ebp1. Example of the four cytoplasmic staining intensities for Ebp1: 0+, 1+, 2+ and 3+. B) Median staining intensity of Ebp1 in normal (n = 39), normal adjacent (n = 53), PIN (n = 29), tumor (n = 62) and hormone refractory (n = 36) prostate tissues. Normal tissues come from the N-TMA. Normal Adjacent, PIN and tumor tissues come from the RP-TMA. The hormone refractory tissues come from the HR-TMA. Significant statistical differences were found Normal vs Normal Adjacent Tissues (*P *< 0.001, Mann-U), Normal Adjacent vs Tumor Tissues (*P *< 0.001, Mann-U), PIN vs Tumor (*P *= 0.003, Mann-U) and Tumor vs Hormonorefractory (*P *< 0.001, Mann-U). However, no statistical difference was found between Normal Adjacent vs. PIN (*P *= 0.464, Mann-U). C) Median staining intensity of Ebp1 according to Gleason score. The tissues analyzed are from the tumor cores of the RP-TMA. No significant differences were observed (Mann-U).

**Figure 3:**
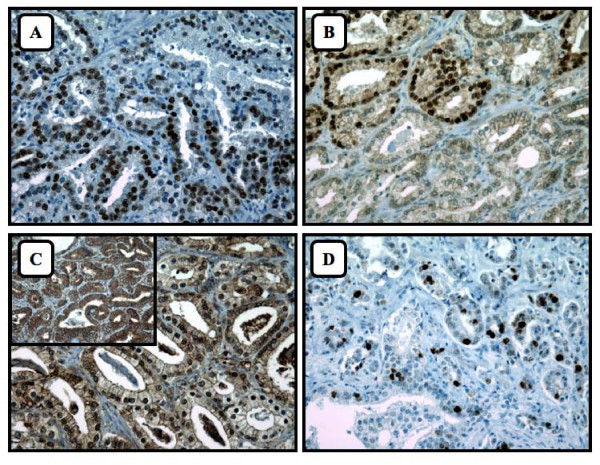
**Expression of individual markers in prostate cancer tissue**. A) Nuclear staining of androgen receptor. B) Nuclear staining of Cyclin D1. C) Nuclear and cytoplasmic staining of ErbB3. Inset: Example of a tissue without nuclear ErbB3 staining. D) Nuclear staining of Ki67.

TMAs were evaluated through a 20× objective using a bright field microscope. For the Ebp1 expression analysis, each core was given a score based on the cytoplasmic staining intensity from 0 to 3 (Figure 2a). For AR, Cyclin D1 and ErbB3, each core was given a score based on the percentage of cells showing positively stained nuclei (Figure 3). Two independent observers analyzed each TMA in a blinded manner. Inter-observer scoring correlation was 0.75 to 0.9. The average of both observations was reported for statistical analysis. The quantification of Ki67 expression was completed with an image analysis software, as previously described by our group, due inherent staining properties, which, unlike the AR, Cyclin D1 and ErbB3, Ki67 strictly localizes to the nucleus [18]. Briefly, a digital image of each core was analyzed with the Image Pro Plus v.5.0 software (MediaCybernetics) and a score representing the number of Ki67 positive (+) nuclei was obtained. The average number of both tumor cores, and the value for the normal core, was used for statistical analysis. Since ROC analyses did not give statistically significant results, data for all five markers were categorized according to the median staining expression. This median was used as a cut-offs for each marker in order to create multi-marker combinations.

### Statistics

Statistical analysis was performed using with SPSS software 11.0 (SPSS Inc. Chicago, Illinois, USA). The median score of both normal adjacent tissues and tumor tissues were used as cutoff values, thus giving two independent scores for each marker: i.e. a normal adjacent value and a tumor value. Since not all marker expression had normal distribution and equal variance, the non-parametric Mann-Whitney *U *test was used to show significant differences between the normal, normal adjacent, PIN, tumor and HR groups. Correlations between markers were done using the non-parametric Spearman's rank correlation coefficient. The median marker expression was used to categorize data in dichotomized variables (positive or negative). The median value in normal adjacent tissues was used to categorize normal adjacent expression and the median value in tumor tissues was used to categorize tumor expression. This approach has been demonstrated to be statistically reliable [19,20]. Marker combinations were determined using these dichotomized values. For example, the combined value "Ebp1+/Cyclin D1- in normal adjacent" was created based on a positive Ebp1 expression (above normal adjacent median) and negative Cyclin D1 expression (below normal adjacent median). The Wald test was used for Cox proportional hazards regression analysis in univariate and multivariate models. The data was tested and met the assumptions for using Cox test. Gleason score (< 7, 7, > 7), pre-operative PSA levels (as a continuous variable), surgical margin status and pathological stage were included in the multivariate analyses as clinical parameters known to correlate with PSA relapse.

## Results

### Ebp1 expression correlates with the presence of prostate cancer

The expression of Ebp1 was analyzed by immunohistochemistry in a total 138 prostates from patients with or without prostate cancer. In the 138 patients analyzed, we did not observe any nuclear localization of Ebp1. The cytoplasmic expression of Ebp1 was therefore studied. We observed a progressive increase in the cytoplasmic staining intensity of Ebp1 from normal tissue of patients with no prostate cancer to the more malignant hormone refractory tumors (Figure 2b). We also observed an increase in Ebp1 expression in tumor tissue compared to normal adjacent tissue (*P *< 0.001, Mann-U). Interestingly, there was a significant difference in the expression of Ebp1 between normal (from patients without prostate cancer) and normal adjacent tissues (from patients with prostate cancer) (*P *< 0.001, Mann-U). There was also a significant increase in the expression of Ebp1 in hormone refractory tumors compared to hormone sensitive tumors (*P *< 0.001, Mann-U). We further evaluated whether Ebp1 expression correlated with the Gleason score of hormone sensitive tumors (Figure 2c). No significant differences in Ebp1 expression were found between Gleason scores (*P *> 0.05, Mann-U).

### Individual expression of Ebp1 does not predict PSA relapse

The prognostic role of Ebp1 with regards to the PSA relapse following radical prostatectomy was evaluated in COX univariate regression models on the RP-TMA (Table 2). We observed that the individual expression of Ebp1 in both normal adjacent and tumor tissues was unable to predict PSA relapse (*P *= 0.170 and *P *= 0.689, respectively).

**Table 2:** Analyses of overall PSA relapse*.

	**Univariate analyses**^†^	
	
	**Normal adjacent expression**	Tumor expression
	Hazard ratio (95% CI);	Hazard ratio (95% CI);
	p-value	p-value
	Cut-off (median)^‡^	Cut-off (median)^‡^
Ebpl	1.75(0.79–3.89);	1.15(0.58–2.26);
	p = 0.170	p = 0.689
	Median = 2.00	Median = 2.50

Androgen Receptor	**0.48 ((0.24–0.95);**	0.51 (0.25–1.02);
	**p = 0.035**	p = 0.056
	**Median = 61.25**	Median = 81.67

Cyclin D1	**0.34(0.17–0.69);**	0.57(0.29–1.14);
	**p = 0.002**	p = 0.113
	**Median = 2.50**	Median = 14,38

ErbB3	**0.42 (0.21–0.85);**	1.16(0.59–2.29);
	**p = 0.016**	p = 0.665
	**Median = 3.75**	Median = 5.63

Ki67	1.03 (0.52–2.04);	1.01 (0,52–1,96);
	p = 0.936	p = 0.976
	Median = 29.50	Median = 127.25

Correlation between PSA relapse and the expression of the five molecular marker expression in normal adjacent and tumor tissues using univariate Cox regression analyses. The median value used as the statistical cut-off is also shown.

* Data was categorized according to the median.

^†^Univariate COX regression with the "ENTER" model

^‡^Median expression was used as cut-offs for catergorized data.

Bold indicates statistically significant values

### Expression of Ebp1-associated proteins correlates with PSA relapse

Since the expression of Ebp1 correlated with prostate cancer progression, we also evaluated whether the expression of Ebp1 correlated with the nuclear expression of Ebp1 regulated proteins (AR, Cyclin D1 & ErbB3) and the proliferation marker Ki67. The immunohistochemical staining for these four proteins was completed on the RP-TMA (Figure 3). Contrary to previously published *in vitro *data describing that Ebp1 can inhibit the expression of AR, our *in vivo *results illustrated that cytoplasmic Ebp1 positively correlated with AR nuclear expression in normal adjacent (Spearman = 0.295, *P *= 0.032) and tumor tissues (Spearman = 0.374, *P *= 0.003) (Table 3). We observed that Ebp1's cytoplasmic expression also correlated strongly with the nuclear expression of Cyclin D1 and ErbB3 in both normal adjacent and tumor tissues (Table 3).

**Table 3:** Correlation between the expressions of individual markers*.

		**Normal adjacent Expression**					**Tumor expression**				
			
		**AR **Spearman; P-Value	**CyclinD1 **Spearman; P-Value	**Ebp1 **Spearman; P-Value	**KrbB3 **Spearman; P-Value	**Ki67 **Spearman; P-Value	**AR **Spearman; P-Value	**CyclinD1 **Spearman; P-Value	**ErbB3 **Spearman; P-Value	**Ebp1 **Spearman; P-Value	**Ki67 **Spearman; P-Value
**Normal Adjacent**	**AR**	-	-	-	-	-	-	-	-	-	-
		-	-	-	-	-	-	-	-	-	-
		
	**CyclinD1**	**0.612**	-	-	-	-	-	-	-	-	-
		**< 0.001**	-	-	-	-	-	-	-	-	-
		
	**Kbp1**	**0.482**	**0.369**	-	-	-	-	-	-	-	-
		**< 0.001**	**0.007**	-	-	-	-	-	-	-	-
		
	**ErbB3**	**0.700**	**0.727**	**0.425**	-	-	-	-	-	-	-
		**< 0.001**	**< 0.001**	**0.002**	-	-	-	-	-	-	-
		
	**Ki67**	**0.326**	**0.307**	**0.362**	0.167	-	-	-	-	-	-
		**0.017**	**0.025**	**0.008**	0.231	-	-	-	-	-	-

	**AR**	**0.504**	**0.493**	**0.258**	**0.482**	0.051	-	-	-	-	-
		**< 0.001**	**< 0.001**	**0.065**	**< 0.001**	0.718	-	-	-	-	-
		
	**CyclinD1**	0.249	**0.370**	0.166	**0.408**	**0.340**	**0,368**	-	-	-	-
		0.075	**0.007**	0.240	**0.003**	**0.014**	**0.004**	-	-	-	-
		
	**ErbB3**	0.169	0.030	**0.323**	0.065	**0.354**	**0.498**	**0.344**	-	-	-
		0.232	0.831	**0.019**	0.646	**0.010**	**< 0.001**	**0.007**		-	-
		
	**Ebp1**	**0.295**	**0.317**	**0.397**	**0.483**	0.190	**0.374**	**0,394**	**0.348**	-	-
		**0.032**	**0.021**	**0,003**	**< 0.001**	0.172	**0.003**	**0.002**	**0.006**	-	-
		
	**Ki67**	**0.310**	0.083	0.247	0.152	**0.436**	**0.256**	0.007	**0.285**	0.064	--
		**0.024**	0.555	0.074	0.276	**0.001**	**0.046**	0.954	**0.026**	0.622	-

Non-parametric correlations between the expressions of the individual markers.

* The correlation between marker expression was analyzed with continuous, non-categorical values.

Bold indicates statistically significant values

Subsequently, we evaluated whether the nuclear expression of AR, Cyclin D1, ErbB3 and Ki67 could predict PSA relapse. The level of nuclear expression of AR, Cyclin D1 and ErbB3 in normal adjacent tissue were all associated with a lower risk of developing PSA relapse in a COX univariate regression model (Table 2). No individual marker nuclear expression in the tumor tissue was associated with PSA relapse.

### Multi-marker combinations are independent predictors of PSA relapse

Due to the strong expression correlation between the five molecular markers, we evaluated whether double and triple marker combinations could predict PSA relapse in tumor tissues. Contrary to AR, Cyclin D1, ErbB3 and Ki67 nuclear expression, high Ebp1 expression tended to positively correlate with a higher risk of PSA relapse (Kaplan-Meier, data not shown). As such, we studied whether marker combinations composed of elevated Ebp1 and low expression of the four other markers were associated with PSA relapse (e.g. Ebp1+/Cyclin D1-). Of all the various combinations analyzed, 8 combinations were associated with PSA relapse in univariate COX regression models.

Finally, we investigated the predictive strengths of the individual maker expression and the various combinations in multivariate models with a base clinical model composed of: Gleason score (< 7, 7, > 7), pre-operative PSA levels, surgical margin status and pathological stage (Table 4). As previously reported in the literature, univariate analyses of the base clinical model showed that the Gleason score, surgical margin status and P-Stage correlated with PSA relapse [21,22]. In a multivariate analysis, the Gleason score (HR = 4.21, *P *= 0.030) and surgical margin status (HR = 8.06, *P *< 0.001) were found to be independent predictors of prostate cancer recurrence. By adding the molecular markers to the base model, we found that the two marker combinations in the tumor tissue were identified as independent predictors: CyclinD1+/ErbB3+ (HR = 0.44, *P *= 0.034) and Ebp1+/Cyclin D1- (HR = 4.79, *P *= 0.003) (Table 4).

**Table 4:** Univariate and multivariate Cox regression analyses of PSA relapse.

	**Univariate analyses**	**Multivariate analyses**		
		
		**Base model**	**Base model + CyclinD1+/ErbB3+(T)**	**Base model + Ebp1+/CyclinD1-(T)**
				
	Hazard ratio (95% CI); p	Hazard ratio (95% CI); p	Hazard ratio (95%CI); p	Hazard ratio (95%CI); p
Gleason < 7 vs Gleason 7	1.67	0.99	0.96	1.36
	(0.78–3.57);	(0.45–2.19);	(0.43–2.17);	(0.58–3.20);
	p = 0.186	p = 0.977	p = 0.924	p = 0.484
Gleason < 7 vs Gleason > 7	2.80	4.21	5.75	6.80
	(1.15–6.81);	(1.65–10.75):	(2.14–15.44);	(2.40–19.30):
	p = 0.023	p = 0.030	p < 0.001	p < 0.001

Pre-Op PSA	1.03	..	..	..
	(0.99–1.07);	..	..	..
	p = 0.198	..	..	..

Surgical Margins	5.97	8.06	9.91	12.06
	(2.68–13.3);	(3.32–19.55);	(3.96–24.81);	(4.48–32.49);
	p < 0.001	p < 0.001	p < 0.001	p < 0.001

P-Stage	5.15	..	..	..
	(2.49–10.63);	..	..	..
	p < 0.001	..	..	..

Molecular Marker	..	..	0.44	4.79
	..	..	(0.20–0.94);	(1.73–13.26);
	..	..	p = 0.034	p = 0.003

Model *P *value	..	p < 0.001	p < 0.001	p < 0.001

Correlation between PSA relapse and the clinico-pathological parameters using univariate Cox regression analyses. Multi-variate Cox regression analysis of the base models and the base model + molecular marker.

* Multi-Variate COX regression was perform under the Forward Wald model

Normal Adjacent Tissues (NA): Tumor Tissues (T)

## Discussion

Prostate cancer continues to represent a serious health problem for aging men in Western countries. Within the patients who are treated by radical prostatectomy, it remains difficult to identify which patients will be at risk for prostate cancer recurrence. Our goal was to characterize Ebp1 expression in prostate cancer tissues obtained from radical prostatectomy and to evaluate whether Ebp1 could represent a molecular marker capable of identifying patients at higher risk of disease recurrence or progression. Our results demonstrate that Ebp1 expression correlated with the progression from normal, to normal adjacent, to hormone sensitive prostate cancer and to hormone refractory prostate cancer. This data is consistent with previously published results demonstrating an overexpression of Ebp1 in colorectal cancer [14]. However, contrary to published *in vitro *data using prostate cancer cell lines [7,11-13,23], no nuclear localized Ebp1 was observed in the human tissue samples that we tested.

Interestingly, normal adjacent tissue expressed higher levels of Ebp1 than normal tissue from patients without prostate cancer. We suspect that the difference in expression of Ebp1 between the two "normal" tissues could be explained by the tumor's field effect on the surrounding cells. Normal adjacent tissue was found to contain a wealth of valuable molecular prognostic information. In fact, expression of the AR, Cyclin D1 and ErbB3 in normal adjacent tissue was found to be more informative than the corresponding tumor tissue in predicting PSA relapse in univariate models.

Strong positive correlations were also found between the cytoplasmic expression of Ebp1 and AR, Cyclin D1 and Erb3. Previous reports have suggested that the anti-proliferative activity of Ebp1 is dependant on its nuclear localization [24]. We found that cytoplasmic Ebp1 positively correlated with the nuclear expression Cyclin D1. There exists two isoforms of Ebp1: the p42 isoform, which inhibits cellular proliferation, and the p48 isoform, which promotes proliferation and cell survival [25]. The antibody used in this study recognizes the p48 and not the p42 isoform. Although the expression of two different Ebp1 isoforms in prostate cancer has not yet been examined, the expression of p42 and p48 isoforms may explain Ebp1's regulation of cellular proliferation in prostate cancer. In line with this hypothesis, we found that high Ebp1 expression correlated with high nuclear AR expression, which is inconsistent with *in vitro *data in LNCaP cells [12]. Similarly, the inhibition of AR expression and AR-regulated genes may be dependant on Ebp1's nuclear translocation. Future work should evaluate the expression of the p42 isoform in prostate cancer.

Furthermore, our results are consistent with results from our group detailing that ErbB3 does not predict overall biochemical recurrence except in patients with positive surgical margins [5]. This study adds to the list of publications suggesting that nuclear AR expression in tumor tissue is non-informative as a prognostic marker in prostate cancer [26-29]. However, a high expression of AR in normal adjacent tissue appears to protect against PSA relapse. This suggests that the AR in normal adjacent tissues may play a different role as compared to the AR in tumor tissue where the high AR expression appears to be implicated in progression to hormonorefractory disease. Studies have demonstrated significant differences in the expression profiles of normal adjacent tissues compared to tumor cells in prostate [30] and lung cancer [31]. Nonetheless, it has not been established whether these differences could be related to disease progression and prognosis. Based on our findings and those of others, prognostic studies with molecular markers should not be restricted to tumor tissue [32].

Due to the heterogeneity of prostate tumors, the use of single markers may only identify a portion of high-risk tumors. The combination of markers could thus overcome this limitation. Clearly our findings on multi-marker combinations as independent predictors of PSA relapse in prostate needs to be confirmed in a larger study. Nonetheless these results suggest that combining biomarkers could significantly improve our current methods of risk stratification based on clinical and pathological characteristics. Several studies have shown that including multiple biological makers with known biological interactions could significantly improve the predictive ability of nomograms solely based on clinico-pathological parameters [20,33-35]. The next step will be to perform these types of analyses on pre-surgery biopsy samples in order to exploit the predictive strengths of molecular markers earlier in the decision-making and in patients undergoing radiotherapy or other non-surgical therapy.

## Conclusion

In this study, we demonstrated that Ebp1 expression increases with the progression from normal to hormone sensitive and to hormone refractory prostate cancer. Although by itself Ebp1 expression did not predict PSA relapse, two double marker combinations were independent predictors of PSA relapse in a multivariate model. Validation of these findings in larger cohorts is now essential for the eventual use of these molecular markers in clinical practice.

## Abbreviations

(Ebp1): ErbB3-Binding Protein 1; (AR): Androgen Receptor; (PSA): Prostate Specific Antigen; (Rb): Retinoblastoma; (TMAs): tissue micro-arrays; (N-TMA): normal-TMA; (TURP): trans-urethral resections of the prostate; (HR-TMA): hormone-refractory TMA; (RP-TMA): radical prostatectomy TMA; (DAB): diaminobenzidine; (HR): Hazard Ratio.

## Competing interests

The authors declare that they have no competing interests.

## Authors' contributions

POG did the statistical analysis and wrote the manuscript. IHK stained and scored the TMA and participated in the design of the study. CLP scored the TMA and participated in the design of the study. PIK did the interpretation of statistical analysis. AMMM did critical revisions of manuscript and gave the final approval of the version to be published. FS did critical revisions of manuscript and gave the final approval of the version to be published.
